# Psychiatric risk gene transcription factor 4 preferentially regulates cortical interneuron neurogenesis during early brain development

**DOI:** 10.7555/JBR.36.20220074

**Published:** 2022-07-28

**Authors:** Yuanyuan Wang, Liya Liu, Mingyan Lin

**Affiliations:** 1 State Key Laboratory of Reproductive Medicine, Nanjing Medical University, Nanjing, Jiangsu 211166, China; 2 Department of Neurobiology, School of Basic Medical Sciences, Nanjing Medical University, Nanjing, Jiangsu 211166, China

**Keywords:** transcription factor 4, hMGEOs, cortical interneuron, ChIP-seq, neurogenesis, schizophrenia

## Abstract

Genetic variants within or near the transcription factor 4 gene (*TCF4*) are robustly implicated in psychiatric disorders including schizophrenia. However, the biological pleiotropy poses considerable obstacles to dissect the potential relationship between *TCF4* and those highly heterogeneous diseases. Through integrative transcriptomic analysis, we demonstrated that *TCF4* is preferentially expressed in cortical interneurons during early brain development. Therefore, disruptions of interneuron development might be the underlying contribution of TCF4 perturbation to a range of neurodevelopmental disorders. Here, we performed chromatin immunoprecipitation sequencing (ChIP-seq) of TCF4 on human medial ganglionic eminence-like organoids (hMGEOs) to identify genome-wide TCF4 binding sites, followed by integration of multi-omics data from human fetal brain. We observed preferential expression of the isoform TCF4-B over TCF4-A. *De novo* motif analysis found that the identified 5916 TCF4 binding sites are significantly enriched for the E-box sequence. The predicted TCF4 targets in general have positively correlated expression levels with *TCF4* in the cortical interneurons, and are primarily involved in biological processes related to neurogenesis. Interestingly, we found that TCF4 interacts with non-bHLH proteins such as FOS/JUN, which may underlie the functional specificity of TCF4 in hMGEOs. This study highlights the regulatory role of TCF4 in interneuron development and provides compelling evidence to support the biological rationale linking *TCF4* to the developing cortical interneuron and psychiatric disorders.

## Introduction

Transcription factor 4 (TCF4) is a member of the basic helix-loop-helix (bHLH) transcription factors (TFs) family that recognizes an E-box sequence (CANNTG) as homo- or hetero-dimers with tissue-specific bHLH TFs^[1]^. TCF4 is abundantly expressed in several tissues including the brain, and is thought to be involved in neural development processes such as cell proliferation, differentiation, and synaptic formation^[2]^. In addition, TCF4 deficiency, observed in animal model studies, is causal in various cognitive defects^[3]^. Several whole genome association studies have also revealed that *TCF4* is one of the most reproducible susceptibility genes in neurodevelopmental disorders, including schizophrenia (SCZ), Pitt-Hopkins syndrome, autism, and bipolar disorder^[4]^. This suggests there is a link between TCF4 perturbation and neurodevelopmental disorders which must be investigated.

Systematic analyses of expression pattern of TCF4 in the developing and adult mouse brain showed that TCF4 is abundant in germinal regions at early developmental stages *i.e.*, embryonic day 11.5 (E11.5)–E13.5^[2]^. TCF4 expression is also associated with cortical interneuron production timing in medial ganglionic eminence (MGE)^[2,5]^. At E13.5 and after, TCF4 is abundant in the neocortex, corpus callosum, anterior commissure, and hippocampus, and is crucial for brain formation during murine embryonic development^[2,6]^. In addition, ablation of TCF4 during mouse development causes neocortical disorganization, which recapitulates structural brain abnormalities present in Pitt-Hopkins syndrome patients. This evidence suggests that TCF4 may also have a function in normal brain development and neuronal differentiation during embryogenesis^[6]^.

Human neuroblastoma SH-SY5Y cell line, one of the most widely adopted cellular system to model mouse neurodevelopment, has the characteristics of neural stem cells and can differentiate from neuroblast-like state to mature neurons^[7]^. Two recent studies investigated the regulatory role of TCF4 in SH-SY5Y and found that TCF4 target genes were enriched in functional clusters such as ion transportation, signal transduction, and nervous system development^[8–9]^. However, findings from SH-SY5Y studies are unlikely to reflect the relevant cell type-specific role of TCF-4 in neurodevelopment. Fortunately, the revolutionary technology of single-cell transcriptomics has successfully described the landscape of cell types throughout early human brain development. This foundational research helps to identify the most relevant cell types of TCF4^[10–11]^.

To understand the link between TCF4 and psychiatric disorders (PSD), we first conducted an integrative transcriptomics analysis on public bulk RNA-Seq and single-cell RNA-seq (scRNA-seq) data of the fetal brain to determine the spatiotemporal expression pattern of TCF4. We then performed chromatin immunoprecipitation-sequencing (ChIP-seq) on human medial ganglionic eminence-like organoids (hMGEOs) to depict the gene regulatory network of TCF4. To further explore the regulatory mechanism of TCF4 and its relevance to PSD, we conducted an advanced multi-omic analysis integrating landscapes of histone modification, co-expression patterns, and profiles of genetic risks of PSD. Our study suggested interaction with FOS/JUN might determine the functional specificity of TCF4 in hMGEOs, highlighting a potential link between dysregulation of interneuron development induced by TCF4 perturbation and PSD.

## Materials and methods

### Cell culture

The human induced pluripotent stem cell lines (NC3-1, passage 13; ihtc-03, passage 16) were presented by Dr. Yan Liu's laboratory at Nanjing Medical University, China. All stem cell lines were maintained on vitronectin-coated plates (Life Technologies, USA) with Essential eight medium (Life Technologies), and changed daily at 37 °C in 5% CO_2_. Cells were passaged every 5 days through ethylenediaminetetraacetic acid digestion (Lonza, USA).

The human neuroblastoma SH-SY5Y cell line at passage 4 was a gift from Dr. Jun Gao's laboratory (Nanjing Medical University). SH-SY5Y cells were cultured in DMEM/F12 medium (Gibco, USA) supplemented with 10% (v/v) fetal calf serum (Gibco) at 37 °C in 5% CO_2_.

### Development of human medial ganglionic eminence-like organoids

HMGEOs were generated following the protocol developed by Liu *et al*^[12]^, who established a system of directed differentiation for forebrain γ-aminobutyric acid (GABA) interneurons using human induced pluripotent stem cells. After four weeks of differentiation using an established system, more than 90% of the cells become NKX2-1 and FOXG1 expressing MGE progenitors^[12]^.

Stem cells were detached by dispase (Life Technologies) to form embryoid bodies (EB) and then cultured in the neural induction medium (NIM), containing 490 mL DMEM/F12 medium, 5 mL of minimum essential medium non-essential amino acids (MEM-NEAA) (Gibco), 5 mL of N2 supplement (Gibco) for 7 days. Half the NIM medium were changed out every day from day 1 to day 6. After floating for 7 days, EBs were attached on vitronectin-coated surfaces. Rosette structures could be formed during the period from day 10 to day 15. Half the NIM medium were changed out every other day from day 10 to day 15. On day 16, neuroepithelial-containing rosette clones were detached and neuroepithelial cells gradually formed neurospheres. Neurospheres were continuously floated in NIM, and changed half the NIM medium every other day. For ventral differentiation, 500 nmol/L smoothened agonist (SAG, Millipore, Germany) was added from day 10 to day 40. From day 0 to day 10, BMP inhibitor DMH1 (Tocris Bioscience, UK) and TGF-β inhibitor SB431542 (StemGent, USA, Cat. No. 04-0010) were added. Properly developed neurospheres were collected on day 26–40 for follow-up ChIP experiments. The protocol for generating dorsal cortical-like organoids was largely the same except that SAG was not added.

### Immunostaining

First, hMGEOs were fixed with 4% paraformaldehyde (Sangon Biotech, China) for 2 hours in an Eppendorf tube before washing them with phosphate buffered saline (PBS, Beyotime, China) 3 times. Then, organoids were soaked with 20% sucrose (Sangon Biotech) in PBS overnight at 4 °C and then with 30% sucrose in PBS after organoids sinking to the bottom of Eppendorf tube. Organoids were embedded at an optimal cutting temperature compound and cryosectioned at 10 μmol/L prior to immunostaining.

For immunohistochemistry, we blocked and permeabilized each section in 1% Triton (Biolink, China) and 5% donkey serum (Millipore) in PBS before incubating at 4 °C overnight in primary antibody and then in secondary antibody diluted in 5% donkey serum for 1 hour at 20 °C. After performing three 10 minutes washes in PBS, coverslips were mounted for fluorescent imaging using an Eclipse 80i Fluorescence Microscope.

### Western blotting

TCF4 expression in hMGEOs and human dorsal cortical-like organoids (hCOs) was assessed by Western blotting. Organoids were lysed in RIPA buffer (Beyotime) containing protease (Millipore) and a protease inhibitor cocktail (Millipore), then centrifuged at 12 000 *g* for 5 minutes to collect the protein supernatant. Proteins were quantified by BCA Protein Quantitation Kit (Beyotime). After the quantification, proteins were loaded onto gels (Beyotime) separated by SDS-PAGE with 100 V electrophoresis. Then, proteins were transferred onto polyvinylidene fluoride membranes (PVDF) membranes (Millipore) at 300 mA for 2 hours and blocked with 5% (w/v) nonfat dried milk for 2 hours at room temperature. Primary anti-TCF4 (Santa Cruz Biotechnology, USA, Lot No. sc-393407X; dilution 1:1000) antibody was incubated overnight at 4 °C. Anti-GAPDH (Bioworld, China; dilution 1:1000) was used as an internal reference. Then, the membranes were washed with 8× PBST (Beyotime) solution 5 times for 10 minutes and incubated in HRP-conjugated IgG secondary antibody (Biosharp, China; 1:5000 dilution) on a shaker for 2 hours at room temperature. After the incubation, the secondary antibody was decanted, and the membranes were again washed 5 times with 1× PBST for 10 minutes. The enhanced chemiluminescence system (Tanon, China) was used detection of the protein bands.

### Single-cell RNA-seq re-analysis of human medial ganglionic eminence-like organoids and cortical-like organoids

Single-cell expression matrices of human medial ganglionic eminence-like organoids (Day 30 and Day 72, H1 human ES cells, and human iPSC 1090) and cortical-like organoids published by Xiang*et al* were re-analyzed. Expression matrix was processed with Seurat (version 3.1.5)^[13]^. The criteria to select cells for subsequent analysis were as follows: unique molecular identifiers per cell >500, detected genes >300, and a mitochondrial transcript proportion <0.3.

SCTransform normalization^[13]^ was applied to each Seurat object to control confounding sources of variations such as sequencing depth and mitochondrial fraction. Integration was performed to correct batch effect. Visualization of transcriptomic profiles were conducted by uniform manifold approximation and projection (UMAP).

Expression matrices were summarized by the top 10 principal components. The Louvain modularity optimization algorithm was implemented to iteratively group cells into clusters. Cell clusters were annotated to known biological cell types using canonical cell marker genes.

### Chromatin immunoprecipitation-sequencing analysis

ChIP assay was conducted on hMGEOs derived from ihtc-03/NC3-1 and SH-SY5Y cell lines. According to the manufacturer's instructions, libraries of ChIP DNA were prepared using the ChIP Kit (Millipore, Lot No. 17-10086). ChIP assay was performed using anti*-*TCF4 antibody (Santa Cruz Biotechnology, USA, Lot No. sc-393407X; dilution 1:1000) and normal mouse IgG (Millipore, Lot No. 17-10086; dilution 1:1000). We followed ENCODE guidelines for anti-TCF4 antibody validation^[14]^.

To evaluate ChIP enrichment efficiency, we performed real-time quantitative PCR (qPCR) using AceQ qPCR SYBR Green Master Mix (Vazyme, China) for TCF4 binding sites associated with the genes of interests (*SYPL1* [intergenic], *CHRNB4* [intergenic, distal enhancer], *OPRD1* [intron], and *RNU5F-1* [intergenic]) in SH-SY5Y. To evaluate ChIP enrichment efficiency in hMGEOs, we performed qPCR for TCF4 binding sites associated with the genes of interests (*SYT10* [intron], *SEMA3E* [distal intergenic], *CNTNAP2* [distal intergenic], and *BRINP3* [intron]). Values were normalized using the ∆∆Ct method. *GAPDH*was used as an internal reference. Primers were manufactured by Genscript (China). Primer sequences used for qPCR were listed in*** Supplementary Table 1*** (available online).

The sequencing of precipitated DNA of hMGEOs using Illumina Hiseq X-10 (2X150). Sequenced reads were mapped to the human genome (hg38) using BWA software (version 0.7.5a-r405)^[15]^. Only the uniquely mapped reads were retained for further analyses. Peak calling for TCF4 in ihtc-03/NC3-1 was carried out using PeakSeq (version v.1.1, with options "target_FDR 0.05, max_Qvalue 0.05") on the TCF4 ChIP file against the input file and further retains the peak with input reads ≥5 as the significant enrichment peak^[16]^. Genome-wide signal coverage tracks were computed using DeepTools (version 3.3.0, bamCoverage), and visualized in the Integrative Genome Browser (IGV, version 2.8.0)^[17–18]^. Genomic features to peaks were annotated by ChIPseeker (version v1.20.0)^[19]^. The target genes of *TCF4* binding sites were annotated with the Genomic Regions Enrichment of Annotations Tool (GREAT), version 4.0.4, using default parameters^[20]^. *De novo* motifs of ChIP-seq peaks were searched by homer (version v4.11.1) using default parameters^[21]^. Heatmaps of binding signals across multiple genomic locations were drawn by deepTools (computeMatrix command on multiple bigwigs and plotHeatmap), version 3.3.0. GO enrichment analysis and result visualization of TCF4 target genes were performed using enrichplot (version 1.6.1) with R using default parameters^[22]^.

### Upstream regulators analysis

To infer the candidate upstream regulators of the target genes of TCF4 binding sites, we performed upstream regulators analysis by iRegulon, a plugin in Cytoscape, using default parameters^[23]^. The corresponding normalized enrichment score (NES) and the number of regulated genes for each inferred upstream regulator were obtained from iRegulon and visualized in R software.

### TCF4 co-occurring motif combinations analysis

We first extracted the coordinates of the classical motif of TCF4 hit regions within the TCF4 binding sites using MEME mast software and then extended them by 50 bp in both directions^[24]^. These regions were considered likely regions harboring TCF4 and potential co-factors. We conducted SIOMICS (with options "-e 0.00005 -c 0.05") analysis on these extended regions to identify co-occurring motif combinations of TCF4^[25]^. TCF4 binding sites in SH-SY5Y were obtained from a previous report^[8]^, and the TCF4 co-occurring motif combinations in SH-SY5Y were identified as above.

### Psychiatric disorders risk gene enrichment analysis

To determine whether TCF4 targets in hMGEOs were enriched with PSD risk genes, we intersected the identified targets with the risk gene list of *de novo* variants of PSD from Fromer *et al*^[26]^, and performed enrichment analysis using Fisher's exact one-sided test.

### TCF4 and target genes expression correlation analysis

We calculated cell type-specific mean expression within each gestational week based on scRNA-Seq data from human embryonic prefrontal cortex^[10]^.

To analyze correlations between TCF4 and target genes across gestational weeks in cortical interneuron, Pearson correlation analysis was conducted using mean values for TCF4 at nine gestational weeks in cortical interneuron and mean expression values of each target gene of all gestational weeks in cortical interneuron. Target genes with a correlation *P*-value less than 0.05 and a correlation coefficient greater than 0 were positively correlated with TCF4 expression. Conversely, target genes with a *P*-value less than 0.05 and a correlation coefficient less than 0 were negatively correlated with TCF4 expression.

### Statistical analysis

ChIP-qPCR data were reported as mean ± standard deviation (SD). Statistical analysis was performed with a Student's *t*-test in R software. Other statistical tests performed were listed in the respective figure legends or sections of methods.

### Data availability

All public data were available from Gene Expression Omnibus (GEO). The single cell expression dataset from the human embryonic brain was downloaded under the accession number GSE104276^[10]^ and GSE103723^[11]^. The single cell expression dataset from human medial ganglionic eminence-like organoids (Day 30 and Day 72, H1 human ES cells, and human iPSC 1090) and cortical-like organoids were downloaded under the accession number GSE98201^[27]^. The H3K27ac and H3K4me3 ChIP-seq dataset of the human fetal brain were downloaded under the accession number GSE63634^[28]^. The two TCF4 ChIP-seq dataset were downloaded under the accession number GSE96915^[8]^ and GSE112704^[9]^.

## Results

### Validation of TCF4 antibody

**Figure 1:**
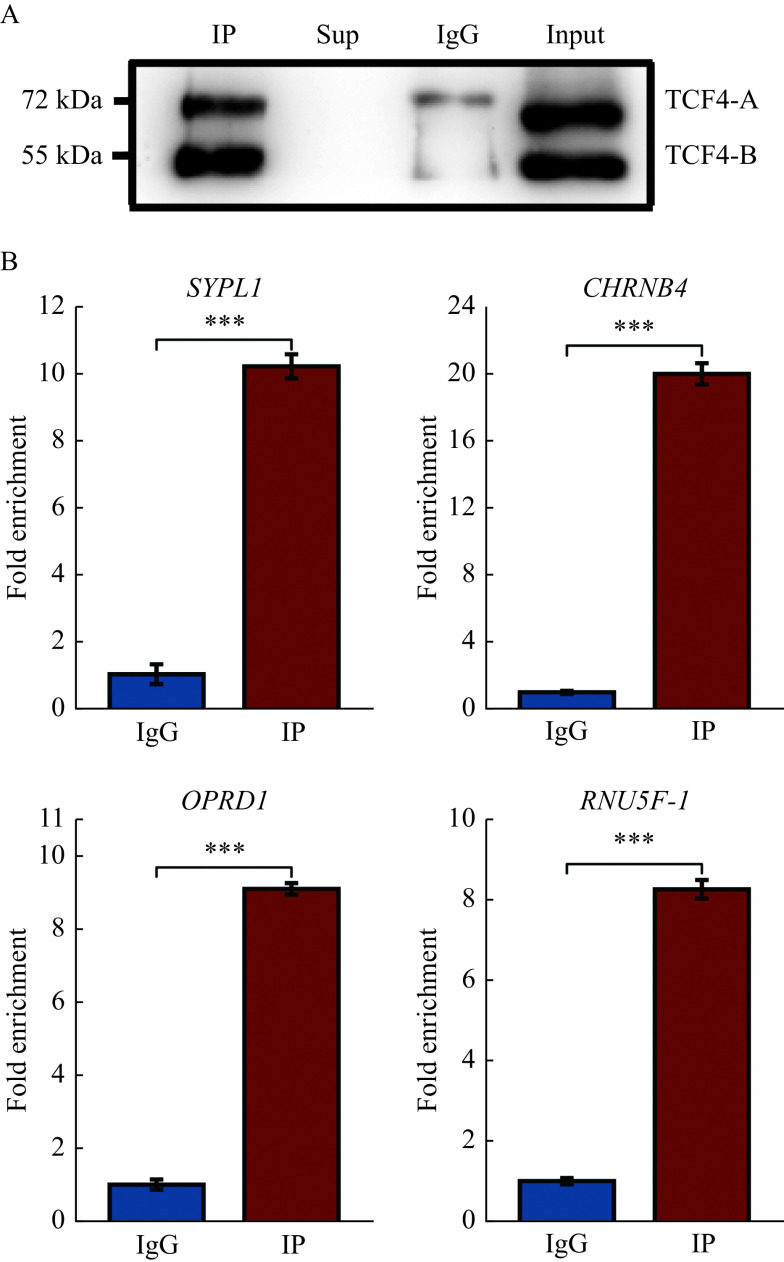
Validation of anti-TCF4 and the efficiency of chromatin immunoprecipitation assay.

The anti-TCF4 antibody used met the specificity and sensitivity quality control criteria for ChIP antibody in ENCODE guidelines^[14]^ and detected the two TCF4 isoforms in SH-SY5Y, TCF4-B (72 kDa) and TCF4-A (55 kDa) (***Fig. 1A***). The previous study by Forrest *et al* have shown that TCF4 regulates gene expression of *SYPL1*, *CHRNB4*, *OPRD1*, and *RNU5F-1*DNA in SH-SY5Y, which was confirmed by our findings that TCF4-bound DNA fragments within or nearby the *SYPL1*, *CHRNB4*, *OPRD1*, and *RNU5F-1* were significantly enriched in our ChIP experiment (***Fig. 1B***).

### Spatiotemporal expression pattern of TCF4

**Figure 2:**
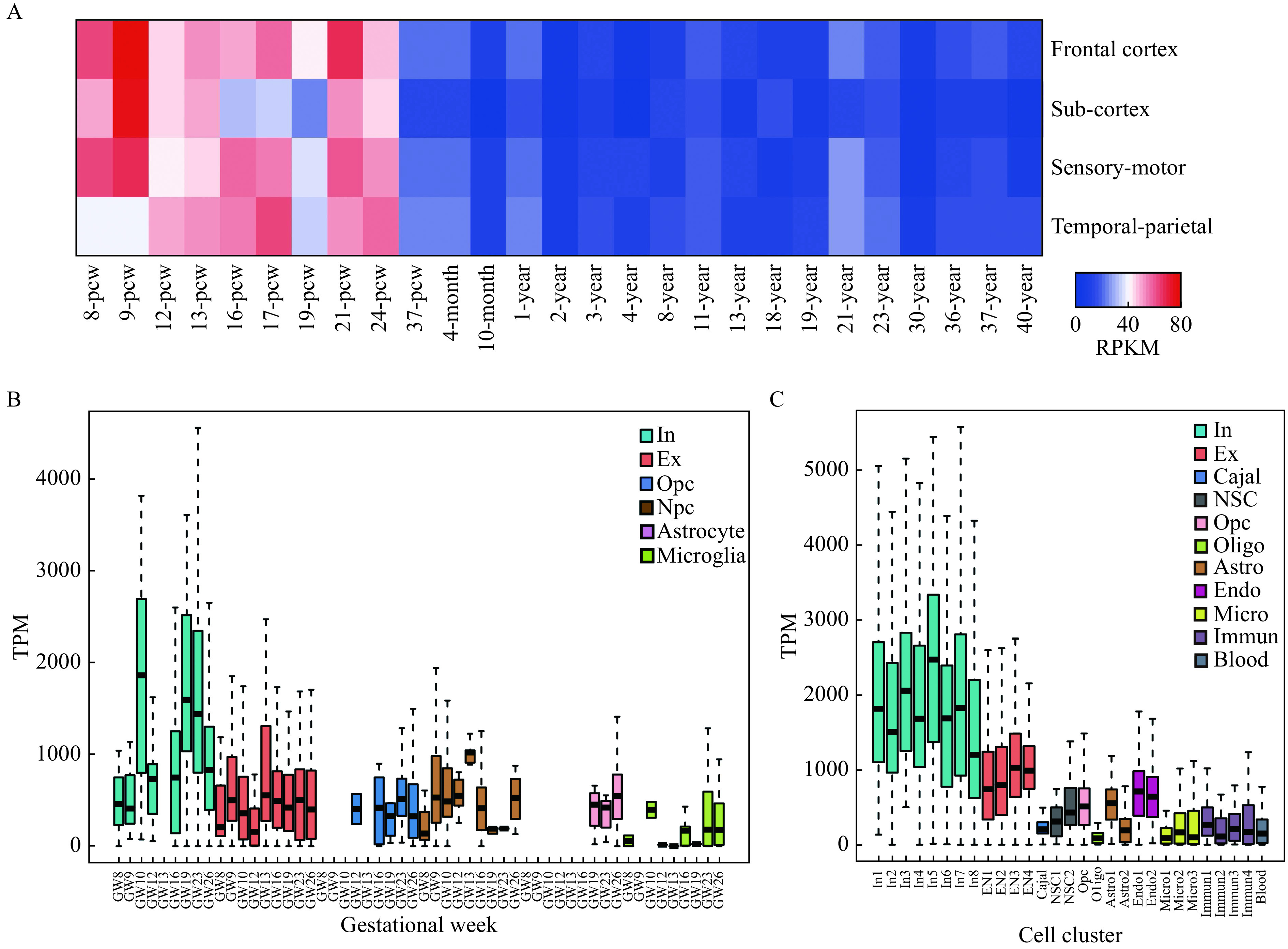
TCF4 is preferentially expressed in cortical interneurons.

To investigate the spatiotemporal expression pattern of TCF4 in the human brain, we leveraged the transcriptional profile of the whole human brain in the BrainSpan data (http://www.brainspan.org/). We found the expression of TCF4 was largely restricted to the prenatal stages of the prefrontal cortex (***Fig. 2A***), suggesting that TCF4 may play a role in the prefrontal cortex during prenatal development.

To identify the most relevant cell type of TCF4 function during neurodevelopment, we re-analyzed scRNA-Seq data from human embryonic prefrontal cortex at gestational weeks (GW) 8 to 26 to quantify the expression level of TCF4 in each cell type^[10]^. The results showed that TCF4 was preferentially expressed in cortical interneuron during early neurodevelopment (***Fig. 2B***). We confirmed this finding by using another set of high-resolution scRNA-Seq data from the entire human cortex at post-conceptional weeks 22 to 23 (22 and 23 W)^[11]^ (***Fig. 2C***). Collectively, these results implied that TCF4 might mainly be involved in interneuron development during early neurodevelopment.

**Figure 3:**
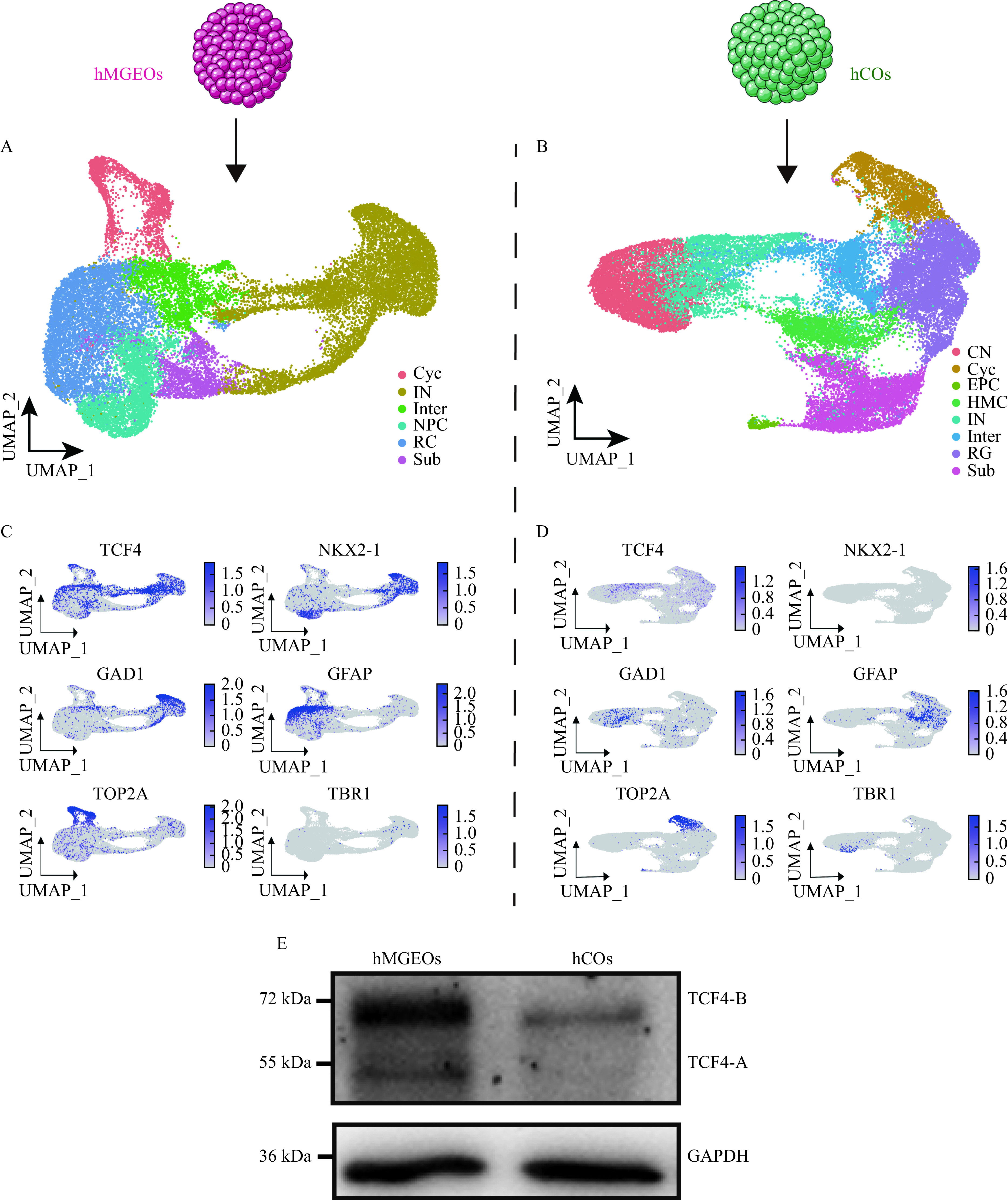
scRNA-Seq analysis showed the expression pattern of TCF4 in hMGEOs and hCOs.

Since interneurons primarily originate from the medial ganglionic eminence (MGE)^[29–31]^, we tested if the hMGEO was suitable for studying the role of TCF4 in fetal interneurons by leveraging single RNA seq data of hMGEOs and hCOs^[27]^. It showed that TCF4 was dominantly expressed in interneurons marked by NKX2-1 and GAD1 in hMGEOs (***Fig. 3A*** and ***C***), while TCF4 could be detected in both interneuron and radial glia in hCOs (***Fig. 3B*** and ***D***). Western blotting revealed that TCF4 was more enriched in hMGEOs compared to hCOs (***Fig. 3E***). In sum, these results suggested the hMGEO was a more desirable model for studying TCF4 and further supported a preferential role of TCF4 in interneurons.

### The functional role and regulatory pattern of TCF4

**Figure 4:**
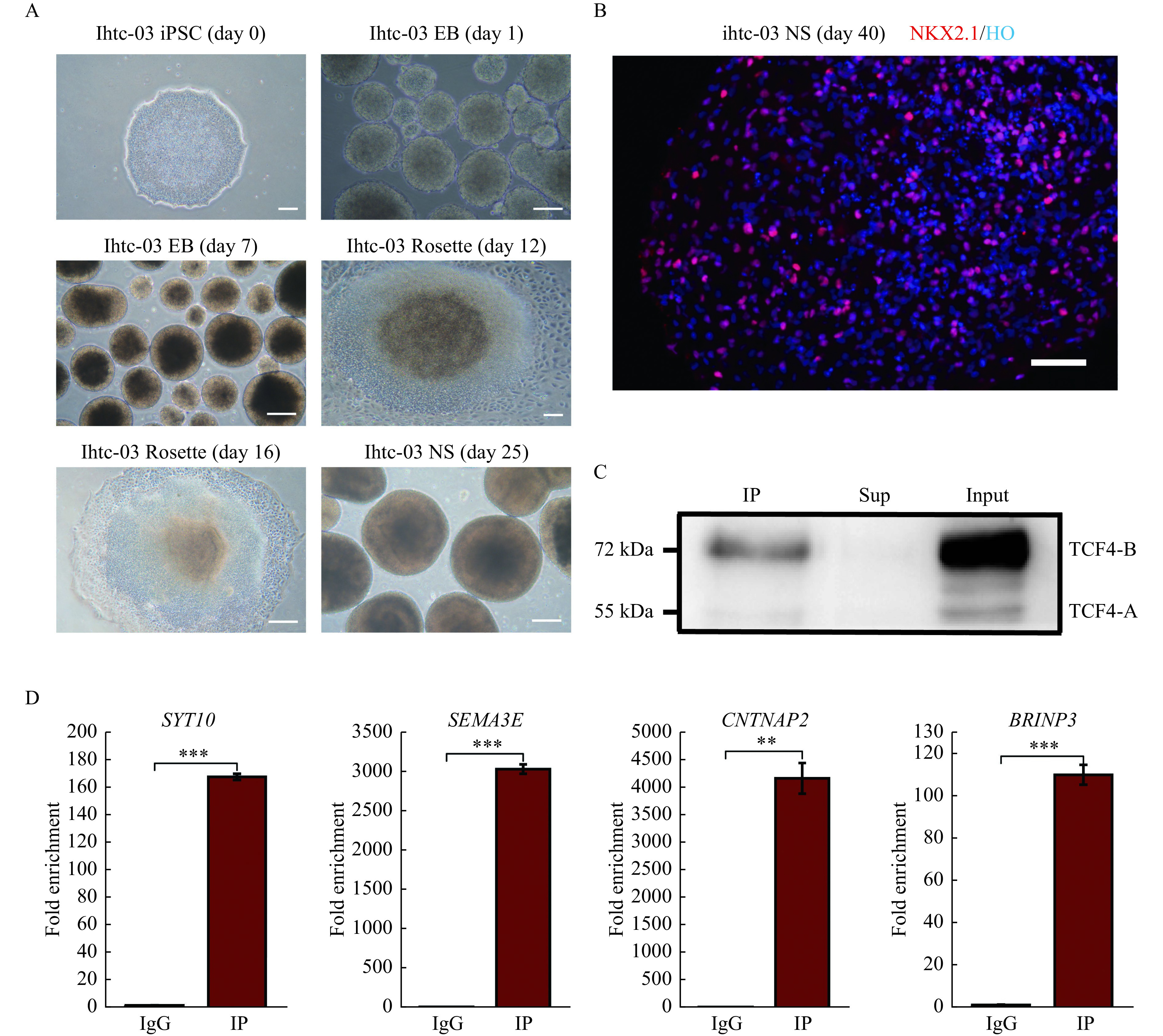
TCF4 ChIP assay in hMGEOs.

Therefore, we developed the hMGEOs following the protocol described previously^[12]^. On day 40 of differentiation, we observed a high level of transcription factor NKX2-1 expression in hMGEOs (***Fig. 4A*** and ***B***). We hypothesized that the genes regulated by TCF4 in hMGEOs could provide functional insight into the role of TCF4 in neurodevelopmental disorders including schizophrenia. To this end, we carried out a ChIP-seq analysis to define the genomic targets of TCF4. Intriguingly, the TCF4-A was barely detected in hMGEOs (***Fig. 3E*** and ***Fig. 4C***). As expected, DNA fragments from the TCF4 binding sites associated with neurogenesis genes such as *SYT10*, *SEM3EA*, *CNTNAP2*, and *BRINP3* were significantly enriched in the hMGEOs ChIP assay (***Fig. 4D***).

**Figure 5:**
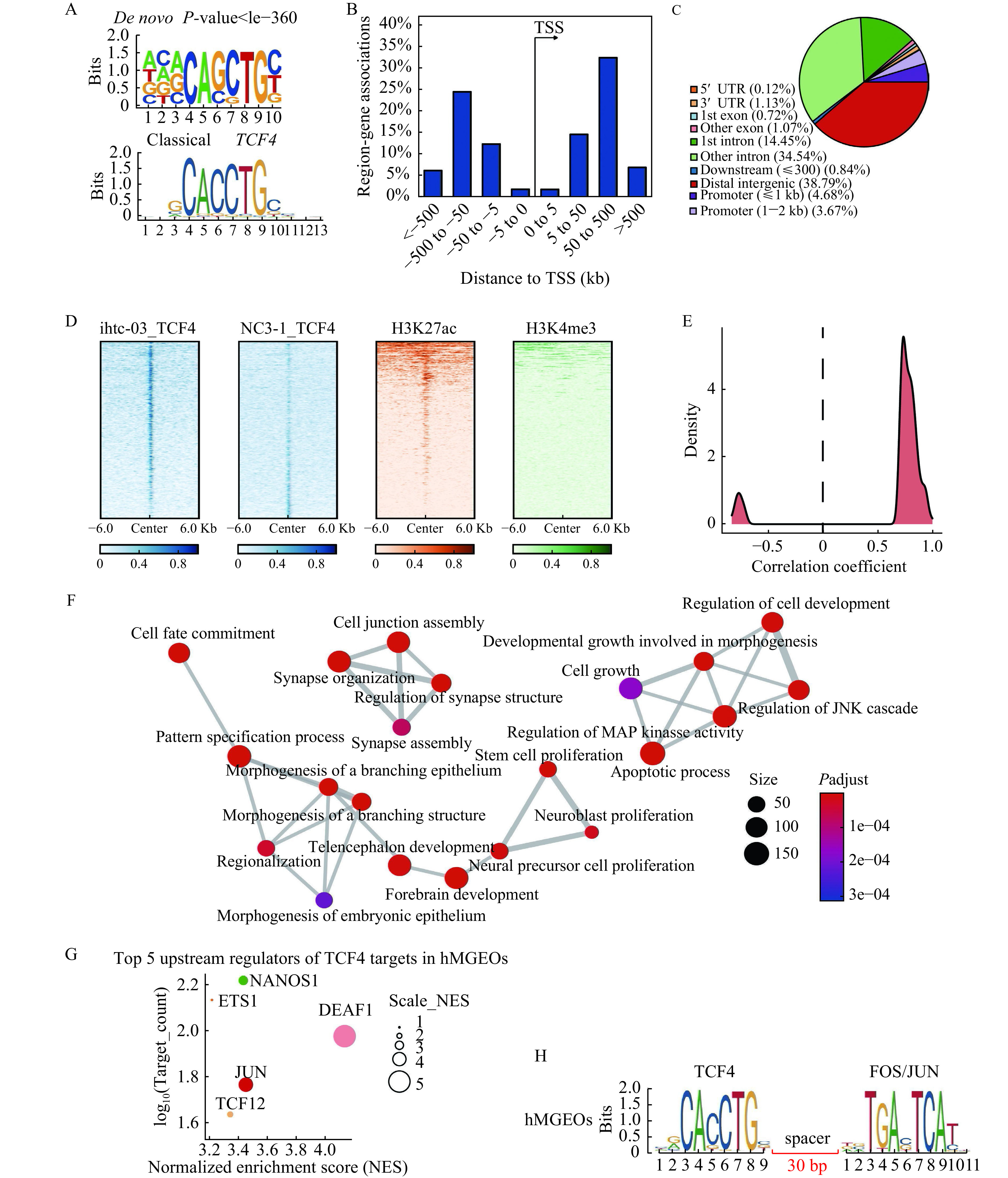
TCF4 primarily activates the transcription of genes associated with neurogenesis by binding to distal enhancers in hMGEOs.

We identified 5916 TCF4 peaks and found that the most enriched *de novo* motif was in high agreement with the classical motif of the bHLH transcription factor families (***Fig. 5A***). To better characterize the functional role and regulatory pattern, we focused on TCF4 peaks containing the classical motif of TCF4 (resulting in 2648 TCF4 binding sites) in the subsequent analysis. We first annotated binding sites and identified target genes using GREAT^[20]^ and found that TCF4 binding sites were enriched at distal genomic regions rather than proximal ones (***Fig. 5B*** and ***C***). In addition, by leveraging publicly available data on active histone modification (H3K27ac and H3K4me3) in the fetal brain at 12 weeks of embryonic development, we showed that the TCF4 binding sites were significantly enriched with the enhancer marker H3K27ac (Fisher's exact *P*-value<4.4×10^−10^, *odds ratio*=1.74) of the early fetal forebrain, but not with the promoter marker H3K4me3 (Fisher's exact *P*-value=0.93, *odds ratio*=0.74) (***Fig. 5D***). These results indicated that TCF4 was bound primarily at the enhancers in hMGEOs during neurodevelopment.

To determine whether TCF4 could activate or repress the transcription of target genes in hMGEOs, we carried out the Pearson correlation analysis between the expression level of TCF4 and 3572 target genes in cortical interneuron based on scRNA-Seq data of the prefrontal cortex during brain development^[10]^. It turned out that a disproportionate amount of target genes was positively correlated with the level of TCF4 expression in cortical interneuron (***Fig. 5E***). These findings, coupled with the observation of the coexistence of TCF4 binding with active enhancer histone modifications, indicated that TCF4 mainly activated transcription distally in hMGEOs during neurodevelopment.

To understand functional clusters of genes regulated by TCF4, gene ontology enrichment analyses were performed on TCF4 targets. These target genes revealed a strong enrichment for neurogenesis events, including neural precursor cell proliferation, apoptotic process, and telencephalon development. (***Fig. 5F***). Notably, TCF4 target genes were significantly enriched for risk genes of neurodevelopmental disorders, including SCZ, autism and intellectual disability (***Table 1***), suggesting TCF4 perturbation could be a major contributor to PSD by regulating many other risk genes at the early stage of neurodevelopment.

**Table 1:** Enrichment of TCF4 target genes for psychiatric risk genes

Gene set	*P*-value^a^	odds ratio^a^	TCF4 bound risk gene count
SCZ_LOF	3.69e−05	2.56	34
SCZ_NS	2.97e−11	1.93	161
SCZ_slient	8.01e−05	1.91	58	
ASD_LOF	3.16e−13	3.96	57
ASD_NS	5.15e−12	1.96	169	
ASD_silent	2.10e−03	1.60	61
ID_LOF	1.11e−11	12.53	23
ID_NS	5.20e−07	3.06	38
ID_silent	2.72e−02	2.75	9
^a^Enrichment for TCF4 target genes in neuropsychiatric gene sets was collated from Fromer *et al*. *P-*values and odds ratio were generated using the Fisher's exact test. *P*-values<0.05 were considered statistically significant. SCZ_LOF: Schizophrenia loss of function mutations genes; SCZ_NS: Schizophrenia nonsynonymous mutations genes; SCZ_silent: Schizophrenia silent mutations genes; ASD_LOF: Autism loss of function mutations genes; ASD_NS: Autism nonsynonymous mutations genes; ASD_silent: Autism silent mutations genes; ID_LOF: Intellectual disability loss of function mutations genes; ID_NS: Intellectual disability nonsynonymous mutations genes; ID_silent: Intellectual disability silent mutations genes.			

Interaction with other factors can influence the regulatory specificities of TCF4 in different cell types. To explore this possibility, first conducting iRegulon analysis to search for upstream candidate regulators of TCF4 target genes in hMGEOs. Among the target genes of TCF4 in hMGEOs,*DEAF1*, *FOS/JUN*, *NANOS*, *TCF12*, and *ETS1* were the most likely upstream regulators (***Fig. 5G***). These candidate upstream regulators may be the downstream targets regulated by TCF4 or the factors cooperating with TCF4. To further infer the interacting factors with TCF4*,* SIOMICS^[25]^ analysis was performed. Notably, transcription factor FOS/JUN involved in neuronal plasticity, neural network formation and immune response were the top TFs co-occurring with TCF4 in the hMGEOs (***Fig. 5H***), suggesting that FOS/JUN might cooperate with TCF4 to regulate interneuron development.

### Difference in the predicted role of TCF4 between hMGEOs and SH-SY5Y

Our study shared less than half of TCF4 target genes with those identified in two previous ChIP-seq experiment of TCF4 in SH-SY5Y^[8–9]^ (***Supplementary Fig. 1A*** and ***B***, available online). Enrichment analysis showed TCF4 target genes in hMGEOs were featured by biological processes involved in neurogenesis (***Fig. 5F***), while target genes in SH-SY5Y converged on biological events related to the capacity of exploring the environment such as response to external stimuli, neuron projection and migration (***Supplementary Fig. 1C*** and*** D***, available online). We also noticed a difference in the annotation of TCF4 binding sites, as TCF4 bound with both active enhancers (overlapping with fetal brain H3K27ac histone modification in Forrest *et al*: Fisher's exact test *P*-value<2.2×10^−16^, odds ratio=7.28; in Xia *et al*: Fisher's exact test *P*-value<<2.2×10^−16^, odds ratio=28.40) and promoters (overlapping with fetal brain H3K4me3 histone modification in Forrest *et al*: Fisher's exact test *P*-value<2.2×10^−16^, *odds ratio*=7.05; in Xia *et al*: Fisher's exact test *P*-value<2.2×10^−16^, *odds ratio*=11.60) in SH-SY5Y while it showed preferential bindings with active enhancers in hMGEOs (***Supplementary Fig. 1E***–***H***, available online). Upstream analysis further suggested a distinct set of TFs co-occurring with TCF4 in SH-SY5Y, including members of bHLH family such as TWIST2 and NEUROD2 (***Supplementary Fig. 1I***–***L***, available online).

## Discussion

Large-scale human genomic studies have led to the identification of an increasingly long list of risk regions and genes associated with PSD, including TCF4, one of a few genes robustly implicated in the genetic aetiology of these diseases^[4]^. This genomic landscape offers unprecedented advantages for the illumination of disease mechanisms but also presents challenges. A key concern is a highly pleiotropic biology encoded by the risk genes. Great efforts have been made to prioritize cells that are fundamental to the genesis of PSD^[32]^, suggesting reductive targets for experimental modeling. In this study, we showed TCF4 is preferentially expressed in cortical interneurons during early neural development, indicating the perturbed biology by TCF4 genetic variants at this particular spatiotemporal point would play an assignable role in neurodevelopmental disorders. Note that the observed expression pattern of TCF4 is consistent with findings in two recent studies revealing abundant expression of TCF4 in migrated interneurons in cortical development^[2,33]^, further corroborating the link between TCF4 and cortical interneurons.

As a first step to elucidate the function of TCF4 in such a particular context, we performed ChIP-seq experiment on hMGEOs, focusing on delineating the role of TCF4 as a TF in this study. We found that the major isoform expressing in hMGEOs is TCF4-B, which can activate transcription to a greater extent than many others as a result of possession of two transcriptional activation domains^[34]^. Indeed, we demonstrated that the predicted target genes with positively correlated expression with TCF4 outnumbered those with the opposite correlation. Not surprisingly, target genes formed functional clusters overrepresented by ontology terms related to interneuron neurogenesis. Intriguingly, target genes exhibited significant overlap with genes previously implicated in schizophrenia, autism and intellectual disability including a number of critical players in the maintenance of E/I balance^[35]^ such as *ERBB4*, *CNTNAP2*, *NRG1*, *TSC1*, *UBE3A*, *CNTNAP4*, and *DISC1*, supporting a convergent role for TCF4 in modulating the known component of the disease risk mechanism^[36]^. These results together suggested TCF4 should play a positive role in promoting generation of cortical interneuron, and TCF4 perturbation could contribute to the development of PSD by E/I imbalance due to defective interneuron neurogenesis.

Perhaps one of the most interesting findings emerging from our study is the interaction between TCF4 and non-bHLH proteins. It is widely accepted that TCF4 exerts its regulatory roles through homodimerization or heterodimerization with the classical bHLH proteins, such as the neurogenin or NeuroD family^[37]^. In neural progenitor cells (NPCs), TCF4 was shown to interact with bHLH TFs, such as NEUROG1/2, ASCL1, and OLIG1/2, to regulate NPCs maintenance and/or differentiation into neurons, oligodendrocytes, and astrocytes during brain development^[4]^. Forrest *et al* and Xia *et al*'s studies in SH-SY5Y also supported the dimerization of TCF4 with other bHLH proteins^[8–9]^. While it is long recognized that other bHLH family members have the potential to cooperate with non-bHLH proteins^[38]^, evidence to support such kind of interaction for TCF4 during neurodevelopment is just beginning to emerge. For example, a recent study provided transcriptomic evidence that TCF4 interacts with non-bHLH proteins like SOX11 in mouse Satb2+ intercortical projection neurons^[39]^. The predicted interaction between TCF4 with non-bHLH proteins FOS/JUN is not entirely unexpected, as other bHLH proteins, such as MYOD, have been shown to cooperate with FOS/JUN family proteins by binding to regulatory elements adjacent to AP-1 sites^[38]^. This interaction is further supported by a prior study showing that expression levels of *FOS* and *TCF4* were highly coordinated in human ventral forebrain spheroids-derived GABAergic interneurons^[33]^. Nevertheless, further experiments are required to verify the interaction between TCF4 and FOS in interneurons.

The demonstrated differences between our ChIP-seq data in hMGEOs and two other ChIP-seq data in SH-SY5Y for TCF4 in terms of genomic binding sites, functional enrichment and interacting partners should be taken with caution. These may reflect the cell type-specific roles of TCF4, but could also be attributed to technical issues we were unable to address in the present study, such as lacks of matched epigenomic profiles from hMGEOs for multi-omic analysis, or more likely antibody-specific bias. The antibody used in Forrest *et al*'s and our study can recognize both TCF4-A and TCF4-B in SH-SY5Y, while that used in Xia *et al*'s study can only detect TCF4-B in SH-SY5Y. Thus, the inferred functional specificity of TCF4 in different contexts based on the difference between our ChIP-seq data in hMGEOs and Forrest *et al*'s ChIP-seq data in SH-SY5Y is supposed to suffer less from antibody-specific bias but more from confounding effects of TCF4-A, while the inference based on the difference between our ChIP-seq data in hMGEOs and Xia *et al*'s ChIP-seq data in SH-SY5Y would be the opposite.

In conclusion, the identification of genome-wide binding sites for TCF4 in hMGEOs sheds a novel insight into the functional role of TCF4 in cortical development. More importantly, our study provided compelling evidence to support the biological rationale linking TCF4 to the developing cortical interneuron and PSD, and represented several interesting hypotheses to be tested in future neurobiological studies. We hope a better characterization of the connections between TCF4 genetic variants and its pleiotropic biology would eventually turn this gene into potentially druggable targets in treating a range of neurodevelopment disorders.
